# The Hope Paradox: A Systematic Review and Meta‐Analysis of the Association Between Hope and the Acquired Capability to Enact Suicide

**DOI:** 10.1111/sltb.70147

**Published:** 2026-11-12

**Authors:** Timothy B. Salguero, Lindsey Fisher‐Fox, Julia L. Smith, Yancey Sizemore, Kevin L. Rand, Melissa A. Cyders

**Affiliations:** ^1^ School of Science, Department of Psychology Indiana University Indianapolis Indianapolis Indiana USA

**Keywords:** acquired capability, hope, positive psychology, suicide

## Abstract

**Introduction:**

Although Snyder's (1994) conceptualization of hope is typically viewed as protective against psychological distress, emerging evidence suggests an association with higher acquired capability for suicide. This meta‐analysis quantified the association between hope and acquired capability, examined differences across core hope components of pathways and agency, and evaluated potential moderators.

**Methods:**

A systematic review of articles published prior to June 2026 was conducted in PsycINFO, PubMed, and Web of Science. Random‐effects models estimated pooled effect sizes and meta‐regression examined age, sex, and race as moderators.

**Results:**

Across nine samples (*N* = 1896), total hope (*r* = 0.23, *p* < 0.001), pathways (*r* = 0.22, *p* < 0.001), and agency (*r* = 0.16, *p* < 0.001) were significantly and positively associated with acquired capability. Agency exhibited significant heterogeneity, and age significantly moderated this association (*p* = 0.003): higher age was associated with stronger, more positive associations.

**Conclusion:**

These findings suggest a paradoxical association between hope and acquired capability. Limited attention to this association underscores the need for more nuanced clinical assessment and intervention. Increasing hope without strengthening adaptive goals or reasons for living may be ill‐advised. Future research should prioritize longitudinal and experimental designs, comprehensive measures of acquired capability, examination of distinct hope components, and assessment of state hope.

## 1 Introduction

Suicide is a major public health concern (Centers for Disease Control and Prevention 2025 [CDC]; World Health Organization 2025 [WHO]), yet its unpredictability remains a persistent challenge (Chu et al. 2017; Joiner et al. 2012; May and Klonsky 2016; Nock, Borges, Bromet, Alonso, et al. 2008; Rudd et al. 1996; Substance Abuse and Mental Health Services Administration, National Survey on Drug Use and Health 2025; Wenzel et al. 2011). Meta‐analytic evidence demonstrates a significant association between higher hope and decreased suicidal ideation and behavior (Ropaj 2022). Higher levels of hope have been consistently linked to suicide‐related constructs, such as lower thwarted belongingness, perceived burdensomeness, and suicidal ideation (Hollingsworth et al. 2016; Umphrey et al. 2021). However, hope's association with acquired capability for suicide (i.e., acquired capability) remains mixed based on a qualitative review (Ropaj 2023), and the association has not yet been quantified meta‐analytically. The goal of this meta‐analysis was to quantify the association between hope and acquired capability, a construct theorized to be essential to the progression from suicidal desire to suicidal action (Joiner 2005; Van Orden et al. 2010).

Acquired capability for suicide is composed of two components: increased tolerance for physical pain and fearlessness of death (Joiner 2005; Van Orden et al. 2010). When suicidal desire is also present and arises from thwarted belongingness and perceived burdensomeness, and a feeling of hopelessness, these components enable individuals to overcome innate self‐preservation instincts to engage in lethal behavior (Joiner 2005; Van Orden et al. 2010). Acquired capability develops through repeated exposure to painful and provocative events, particularly prior suicide attempts and self‐injury (Joiner 2005; Smith and Cukrowicz 2010), leading to habituation to the act of dying (Joiner 2005). This process varies across individuals (Smith and Cukrowicz 2010) and is shaped by both environmental stressors and genetic predisposition (Smith and Cukrowicz 2010; Smith et al. 2012). Although acquired capability reflects relatively stable traits, it can fluctuate with momentary changes in mood, energy, and motivation, especially during transient states, such as intoxication, heightened emotional reactivity, or manic episodes (Law and Anestis 2021; Schuler et al. 2021; Smith and Cukrowicz 2010). Because acquired capability precedes suicidal behavior, it may represent a critical and potentially modifiable risk factor for suicide prevention (Schuler et al. 2021).

Emerging evidence suggests that higher levels of hope may be associated with greater acquired capability, though findings are mixed, likely due to differences across hope components (Ropaj 2023). Hope is conceptualized as being composed of two interrelated components: pathways, the perceived ability to generate goal‐directed routes (i.e., “way‐power”), and agency, the perceived capacity to initiate and sustain motivation to pursue those routes (i.e., “willpower”) (Snyder 1994, 2002; Snyder et al. 1991). Together, these planning and motivational components guide goal‐directed behavior across contexts (Snyder 2002). Snyder (1994, 2002) theorized that the most concerning configuration of hope components involves high agency coupled with low pathways. In this configuration, individuals may become cognitively constrained to a single perceived route of escaping suffering (e.g., suicide), while retaining the motivation to act on it. Under such conditions, hope may take an unconventional and potentially lethal form, in which ending one's life is viewed as the only viable path to relief (Snyder 2002). Thus, pathways may be negatively associated with acquired capability, whereas, agency may be positively associated with it, reflecting their distinct cognitive and motivational functions.

Moderators, such as sex, age, and race, may also account for variation in effect sizes between studies. First, males, who account for the majority of suicide deaths globally (Centers for Disease Control and Prevention, National Center for Injury Prevention and Control 2024 [NCIPC]), report greater pain tolerance (Berkley 1997) and lower fear of death than females (Ribeiro, Witte, et al. 2014) and display greater levels of acquired capability than females (Witte et al. 2012). In addition, sex has been shown to be associated with hope and suicidal behavior, respectively (Hirsch et al. 2012), and sex may obscure the relationship between hope and acquired capability (Mitchell et al. 2015). Second, as one ages, death anxiety decreases (Chopik 2016; Russac et al. 2007; Sinoff 2017), while hope levels peak in middle adulthood (Marques and Gallagher 2017). Additionally, some evidence suggests that hope may differentially affect the relationship between acquired capability and suicide risk in older adults (McLaren et al. 2022). Third, minoritized groups are disproportionately exposed to chronic discriminatory stressors (Bravo et al. 2023), which have been linked to higher acquired capability (Brooks et al. 2020). Higher hope has also been shown to strengthen associations between racial discrimination and depression (Banks et al. 2008). Additionally, hope may influence suicidal outcomes differently among White and Black individuals (Hirsch et al. 2012), and non‐White racial groups have also reported distinct patterns of relationships between hope components and acquired capability (Davidson et al. 2010; O'Keefe and Wingate 2013). Therefore, analyses are warranted to assess if these variables function as moderators of the association between hope and acquired capability and contribute to between‐study heterogeneity.

To date, although there is a previous qualitative review (Ropaj 2023), no meta‐analytic studies have quantified the association between hope and the acquired capability for suicide. Such work is necessary to clarify its direction and magnitude. We conducted a meta‐analysis to (1) quantify the association between hope and acquired capability, (2) examine how the association may differ across agency and pathways, and (3) explore age, sex, and race as potential moderators of this association. Identifying predictors of suicide risk that precede suicidal behavior, such as acquired capability, is necessary to modify and reduce suicide risk.

## 2 Methods

### 2.1 Eligibility Criteria

The inclusion criteria were as follows: (1) quantitative studies published in peer‐reviewed journals in English or translated into English, (2) studies using self‐report measures of either trait and state hope, (3) studies examining constructs that conceptually overlap with hope but are labeled differently, such as grit (Duckworth et al. 2007) or self‐determination (Wehmeyer et al. 2017), (4) studies using self‐report measures of acquired capability, and (5) adult samples (≥ 18 years). The present study focused exclusively on hope and its components, rather than hopelessness, as most work has largely neglected hope by positioning it as the opposite of hopelessness (Grewal and Porter 2007), which oversimplifies the relationship between these constructs and overlooks evidence demonstrating that hope and hopelessness function as related but distinct factors rather than polar opposites (Huen et al. 2015; MacLeod et al. 1993, 1997; Range and Penton 1994). Hopelessness is also more aligned with pessimism than hope (Huen et al. 2015). The quantitative synthesis was limited to adult samples because dominant suicide risk theories and scales were developed and validated in adults, and developmental differences in cognitive, emotional, and neurobiological processes during childhood and adolescence may limit their generalizability to younger populations (Kirshenbaum et al. 2024). The exclusion criteria were as follows: (1) review articles (e.g., systematic reviews, meta‐analyses), theoretical papers, and qualitative studies, (2) studies measuring hopelessness without clear distinction from hope, (3) studies on euthanasia, physician‐assisted suicide, or politically motivated suicide, and (4) studies not examining the relationship between hope and acquired capability or lacking sufficient data for meta‐analysis.

### 2.2 Search Strategy

A systematic search was performed and documented in accordance with the guidelines specified in the Preferred Reporting Items for Systematic Reviews and Meta‐Analyses (PRISMA) checklist (Page et al. 2021). The keywords used were as follows: (hope OR grit OR self‐determination OR agency OR pathways OR goal* OR “positive expectancy”) AND (acquired capability AND suicid* OR “suicide capability” OR “fearlessness about death”). Databases searched were PsycINFO, PubMed, and Web of Science. Eligible articles were published on or before June 15, 2026. EndNote was used to manage citations. Screening and data extraction were conducted in the systematic review manager, Covidence. A PRISMA flow diagram was generated and is presented in Figure 1. The first author inspected all titles and abstracts, followed by full text. All eligible articles were independently coded and reviewed by the fourth author to assess intercoder reliability; any discrepancies were discussed until full consensus was reached (see Higgins et al. 2024).

**FIGURE 1:**
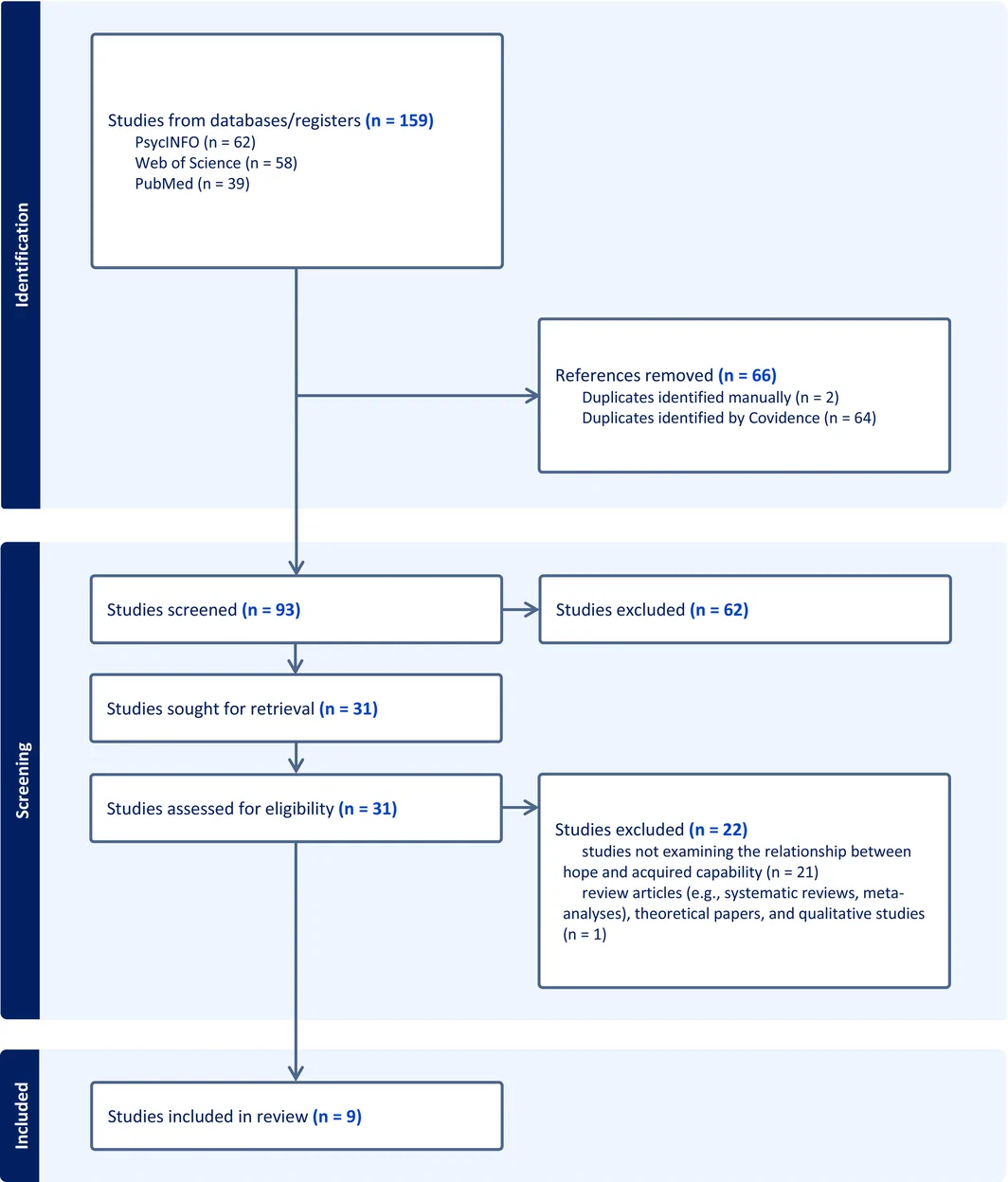
PRISMA flow diagram, illustrating the study screening and selection process. Initial database search was conducted on October 12, 2024 (*n* = 8). An updated search was conducted on June 15, 2026, which yielded one additional record (*n* = 9) and is depicted here.

### 2.3 Data Extraction

Descriptive information for included studies was extracted, including author, year, country, study design, sample type, sample size, mean age, age range, sex (% female), race (% White), measure of hope, measure of acquired capability, and control variables. Effect sizes were extracted for associations of acquired capability with total hope, as well as agency and pathways. Pearson's *r* was prioritized as the effect metric, with standardized beta coefficients (*β*) included when *r* was unavailable (see Peterson and Brown 2005).

### 2.4 Meta‐Analytic Procedures

All analyses were conducted using Comprehensive Meta‐Analysis (CMA) software (Borenstein 2022; Borenstein et al. 2009). Effect sizes were entered directly and weighted by the inverse of their variance. Three analyses were conducted for hope and its subscales (i.e., hope total, agency, and pathways) and their associations with acquired capability using these effect sizes. Random‐effects models were applied to estimate aggregated effect sizes, and when model assumptions differed, following established methodological guidelines (Borenstein et al. 2010). Effect sizes were interpreted using Cohen's (1988) guidelines (i.e., 0.10 represents a small effect, 0.30 a medium effect, and 0.50 a large effect). Heterogeneity among study outcomes was assessed using *I* ^2^ statistics (values of 25% considered low, 50% moderate, and 75% high; Higgins et al. 2003). Publication bias was assessed using funnel plot asymmetry and Egger's regression test (Egger et al. 1997) to evaluate small‐study effects and overall robustness. Sensitivity analyses examined effect size‐type differences (*r* vs. *β*) by excluding studies that used standardized *β* coefficients to evaluate potential bias. Studies reporting statistics that could not be converted to Pearson's *r* or standardized *β* coefficients, or that introduced substantial uncertainty, were excluded.

### 2.5 Moderators

When random‐effects models showed significant heterogeneity (as indicated by the *Q* statistic and *I* ^*2*^ index), moderation analyses were performed. To examine between‐study variability, meta‐regression analyses were conducted using continuous moderators: age, sex (i.e., % female), and race (i.e., % White). These variables were selected based on prior evidence that demographic factors influence acquired capability (Berkley 1997; Chopik 2016; Joiner 2005; NCIPC, 2024; Hirsch et al. 2012; Ribeiro, Witte, et al. 2014; Russac et al. 2007; Mitchell et al. 2015; Marques and Gallagher 2017; Sinoff 2017). Age was coded as the sample's mean age. Race was coded as the percentage of the sample identifying as White. Sex was coded as the percentage of female participants in the sample.

## 3 Results

### 3.1 Study Identification and Selection

Database searches identified 159 relevant articles (see Figure 1). Each database yielded the following: PsycINFO (*n* = 62), PubMed (*n* = 39), and Web of Science (*n* = 58).

Covidence identified and removed 64 duplicate articles through automated screening, and the first author subsequently identified and excluded an additional two duplicate articles through manual review. The total number of duplicates removed was 66, leaving a total of 93 articles eligible for title and abstract review; 62 articles were then identified to be ineligible based on one or more of the criteria identified above. Thirty‐one articles were identified as eligible for full‐text review, 22 of which were excluded, leaving nine articles that met the final inclusion criteria for the review.

### 3.2 Study Characteristics (Table 1)

**TABLE 1:** Study characteristics.

Study	Design	Sample	*N*	Mean age, SD, range	Gender (% female)	Race (% White)	Hope measure	ACS measure	Hope total	Hope agency	Hope pathways	Covariates (when applicable)
Anestis et al. 2014	Cross‐sectional	College	220	*M* = 20.8 SD = 5.3 18–53	77.3%	59.0%	THS	ACSS‐FAD	*r* = 0.23	*r* = 0.15	*r* = 0.27	
Davidson et al. 2009	Cross‐sectional	College	129	*M* = 20.2 — 18–38	65.1%	72.9%	HS‐R2	ACSS	*r* = 0.21	*r* = 0.13	*r* = 0.18	
Davidson et al. 2010	Cross‐sectional	College	115	*M* = 20.4 — 18–31	67.8%	0%	HS‐R2	ACSS	*β* = 0.21	*F* = 1.75	*F* = 2.37	Age, gender, marital status, socioeconomic status
Davidson and Wingate 2013	Cross‐sectional	Psychiatric Inpatient	60	*M* = 26.12 SD = 9.6 18–69	61.7%	73.3%	HS‐R2	ACSS	*r* = 0.27	*r* = 0.19	*r* = 0.21	
O'Keefe and Wingate 2013	Cross‐sectional	College	168	*M* = 23.1 — 18–62	77.4%	0%	THS	ACSS	*r* = 0.23	*r* = 0.20	*r* = 0.23	
McLaren et al. 2022	Cross‐sectional	Community	594	*M* = 68.7 SD = 6.7 60–95	48.8%	96.6%	THS	ACSS‐FAD	*r* = 0.27	*r* = 0.24	*r* = 0.26	
McLaren and Adhikari 2024	Cross‐sectional	Community	300	*M* = 67.1 SD = 6.2 60–90	49.0%	0%	THS	ACSS‐FAD	*r* = 0.16	*r* = 0.19	*r* = 0.11	
Mitchell et al. 2015	Cross‐sectional	College	711	M = 19.4 SD = 2.9 —	67.7%	72.0%	THS	ACSS	—	*r* = 0.05	*r* = 0.23	
Wagshul 2019	Cross‐sectional	Community	193	—	40.4%	80.3%	LOHS	ACSS‐FAD	*r* = 0.20	—	—	

Abbreviations: —, indicates that data were not reported or were insufficient for inclusion; ACS, acquired capability for suicide; ACSS, acquired capability for suicide scale (Van Orden et al. 2008); ACSS‐FAD, acquired capability for suicide scale–fearlessness of death subscale (Ribeiro, Witte, et al. 2014); HS‐R2, revised domain‐specific hope scale (Shorey and Snyder 2004); LOHS, locus of hope scale (Bernardo 2010); THS, trait hope scale (Snyder et al. 1991).

Among the nine studies included, 1896 participants were represented, with individual sample sizes ranging from 60 to 711. The mean age of participants across studies ranged from 19.4 to 68.7 years (*M* = 33.2; SD = 21.5, *k* = 8), with most samples composed primarily of undergraduate college student participants. Two studies exclusively examined older adults (*M* age = 68.7; McLaren et al. 2022; *M* age = 67.1; McLaren and Adhikari 2024), and another reported age distributions rather than a mean (Wagshul 2019), with most participants falling between 21 and 39 years. Sex distribution ranged from 40.4% to 77.4% female (*M* = 61.7%; SD = 13.0%; *k* = 9). Seven studies (77.8%) recruited participants from the United States, while one study recruited its sample from Australia (McLaren et al. 2022), and another recruited its sample from Nepal (McLaren and Adhikari 2024). All studies employed cross‐sectional designs. Five studies used the Acquired Capability for Suicide Scale (ACSS; Van Orden et al. 2008), and four studies used the Acquired Capability for Suicide Scale–Fearlessness of Death subscale (ACSS‐FAD; Ribeiro, Witte, et al. 2014). Five studies used the Trait Hope Scale (THS; Snyder et al. 1991), three studies used the Revised Domain‐Specific Hope Scale (HS‐R2; Shorey and Snyder 2004), and one study used the Locus of Hope Scale (LOHS; Bernardo 2010). Three studies assessed a third hope subscale, “goals” (see HS‐R2; Shorey and Snyder 2004), but it was excluded because one study reported a non‐convertible F statistic, leaving too few studies for reliable meta‐analytic estimation.

### 3.3 Total Hope and the Acquired Capability for Suicide

Eight studies examined the association between total hope and acquired capability, showing consistently small significant positive associations (*N* = 1779). Random‐effects model estimates found an overall small, significant, positive association (*r* = 0.23, 95% CI [0.18, 0.27], *Z* = 9.69, *p* < 0.001; Figure 2A). Publication bias analyses revealed no evidence of small‐study effects. Egger's regression test was non‐significant (*t*(6) = 0.77, *p* = 0.47, 95% CI [−2.41, 1.26]), and the funnel plot displayed a symmetrical distribution of studies, suggesting that selective reporting was unlikely to have influenced the results (Figure 2B). Heterogeneity was negligible (*Q*(7) = 3.03, *p* = 0.88, *I* ^2^ = 0%). A sensitivity analysis testing effect type (*β*) did not meaningfully impact the significance level or effect size (*r* = 0.23, 95% CI [0.18, 0.27], *Z* = 9.43, *p* < 0.001), indicating that the inclusion of the study with a corrected effect statistic did not significantly bias the findings.

**FIGURE 2:**
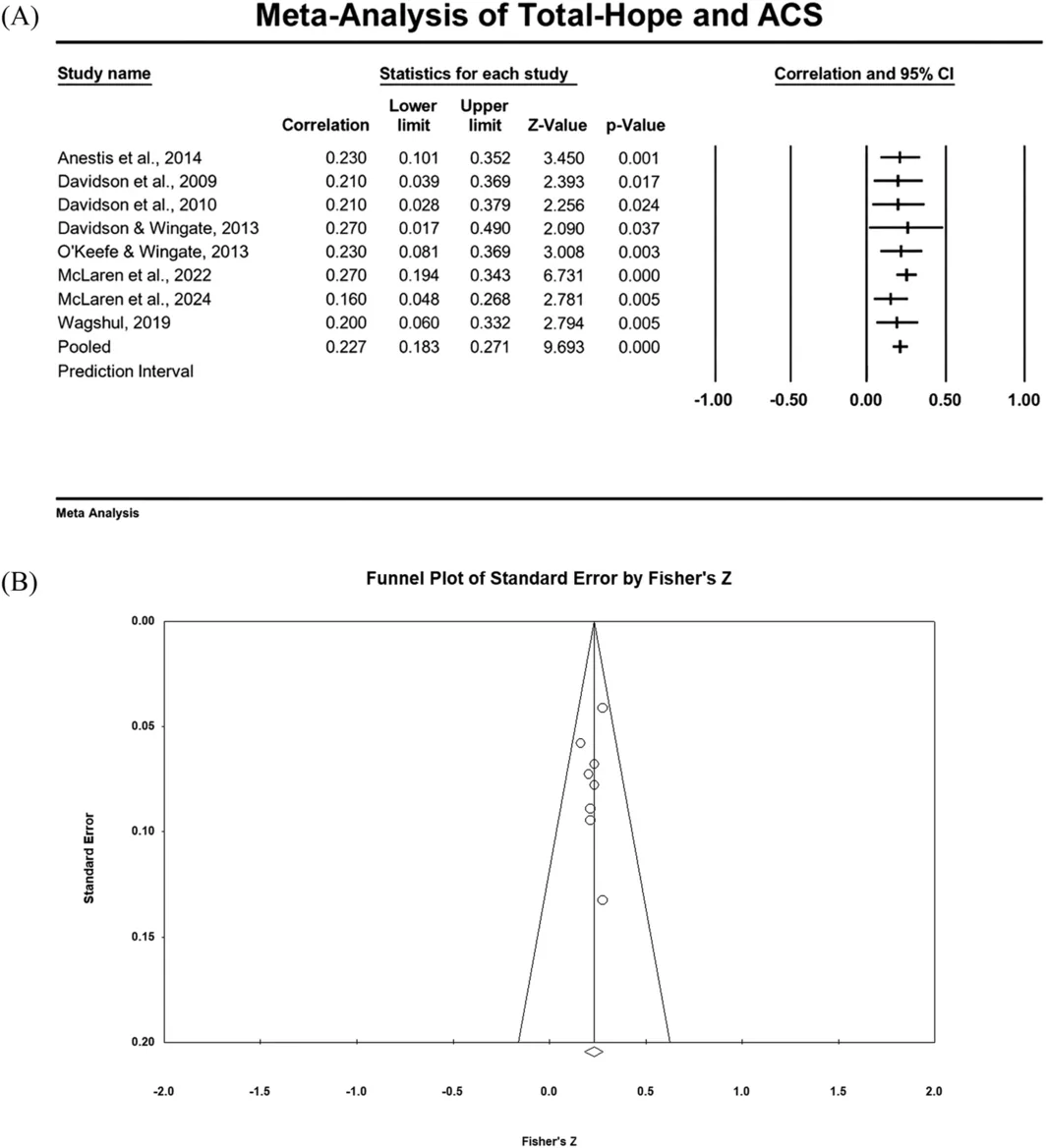
Results for Hope Total. Top panel (A) is the forest plot of the association between hope total and acquired capability for suicide (ACS). Each row represents an individual study association. Points indicate correlation coefficients, horizontal lines represent 95% confidence intervals, and the pooled estimate at the bottom reflects the overall meta‐analytic effect. The bottom panel (B) is the funnel plot assessing publication bias in the association between total hope and acquired capability for suicide. Each point represents a study examining the association between total hope and acquired capability for suicide. Effect sizes are plotted as Fisher's *z* against standard error, with the vertical line indicating the pooled effect estimate.

### 3.4 Hope Pathways and the Acquired Capability for Suicide

Seven studies reported on the association between hope pathways and acquired capability, again showing small significant positive associations (*N* = 2182). Random‐effects model estimates found an overall small, significant, positive association (*r* = 0.22, 95% CI [0.18, 0.26], *Z* = 10.53, *p* < 0.001; Figure 3A). Publication bias analyses revealed no evidence of small‐study effects. Egger's regression test showed no evidence of bias (*t*(5) = 0.60, *p* = 0.57, 95% CI [−3.41, 2.12]), and the funnel plot indicated a balanced distribution of studies (Figure 3B). Heterogeneity was negligible (*Q*(6) = 5.79, *p* = 0.45, *I* ^2^ = 0.00%).

**FIGURE 3:**
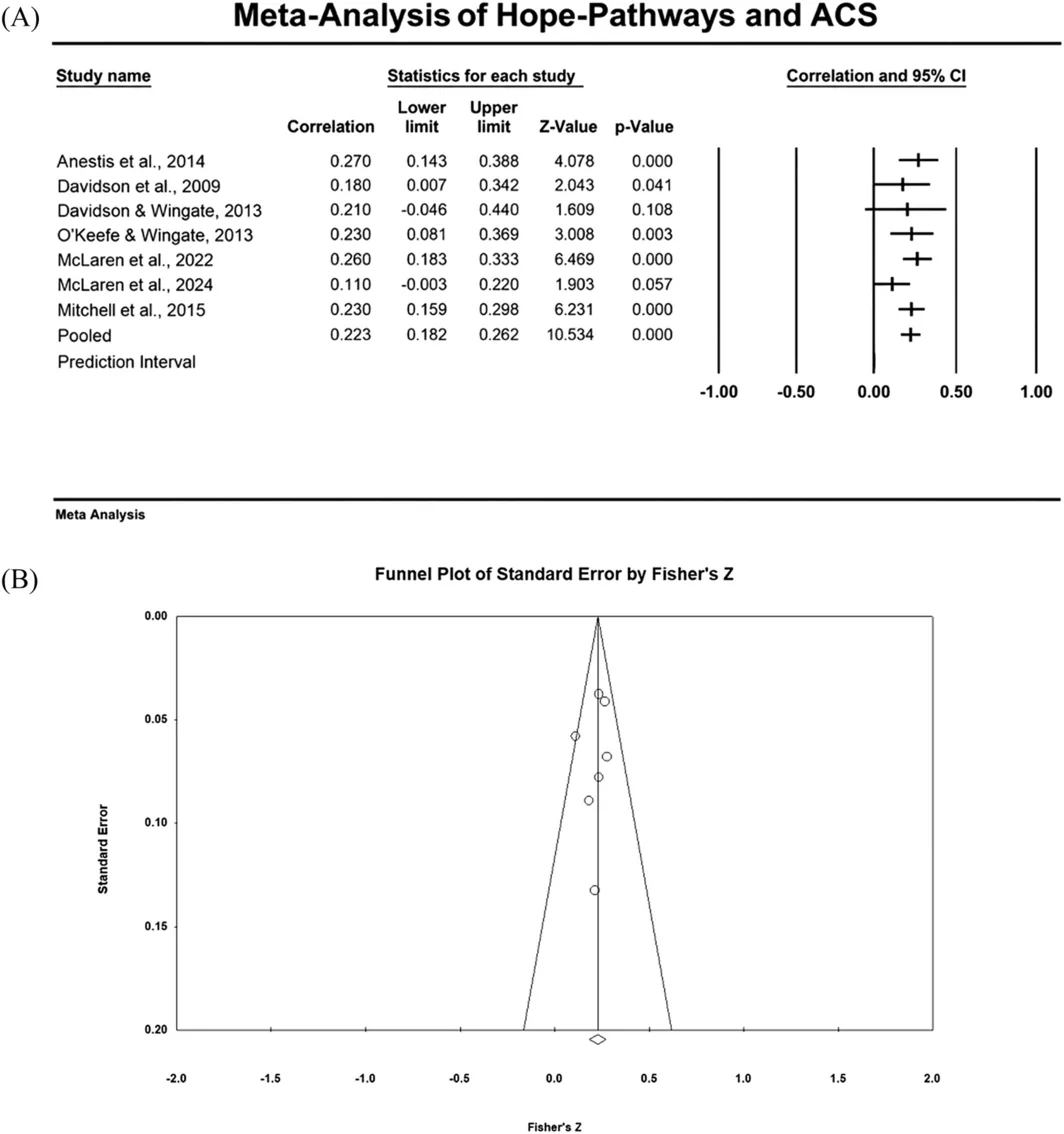
Results for Hope Pathways. Top panel (A) is the forest plot of the association between hope pathways and acquired capability for suicide (ACS). Each row represents an individual study association. Points indicate correlation coefficients, horizontal lines represent 95% confidence intervals, and the pooled estimate at the bottom reflects the overall meta‐analytic effect. The bottom panel (B) is the funnel plot assessing publication bias in the association between hope pathways and acquired capability for suicide. Each point represents a study examining the association between total hope and acquired capability for suicide. Effect sizes are plotted as Fisher's *z* against standard error, with the vertical line indicating the pooled effect estimate.

### 3.5 Hope Agency and the Acquired Capability for Suicide

A total of seven studies reported on the association between hope agency and acquired capability (*N* = 2182). Random‐effects model estimates found a small, significant, positive association (*r* = 0.16, 95% CI [0.09, 0.23], *Z* = 4.55, *p* < 0.001; Figure 4A). Publication bias analyses revealed no evidence of small‐study effects. Egger's regression was non‐significant (*t*(5) = 0.53, *p* = 0.62, 95% CI [−3.38, 5.13]), and the funnel plot showed no visible asymmetry (Figure 4B).

**FIGURE 4:**
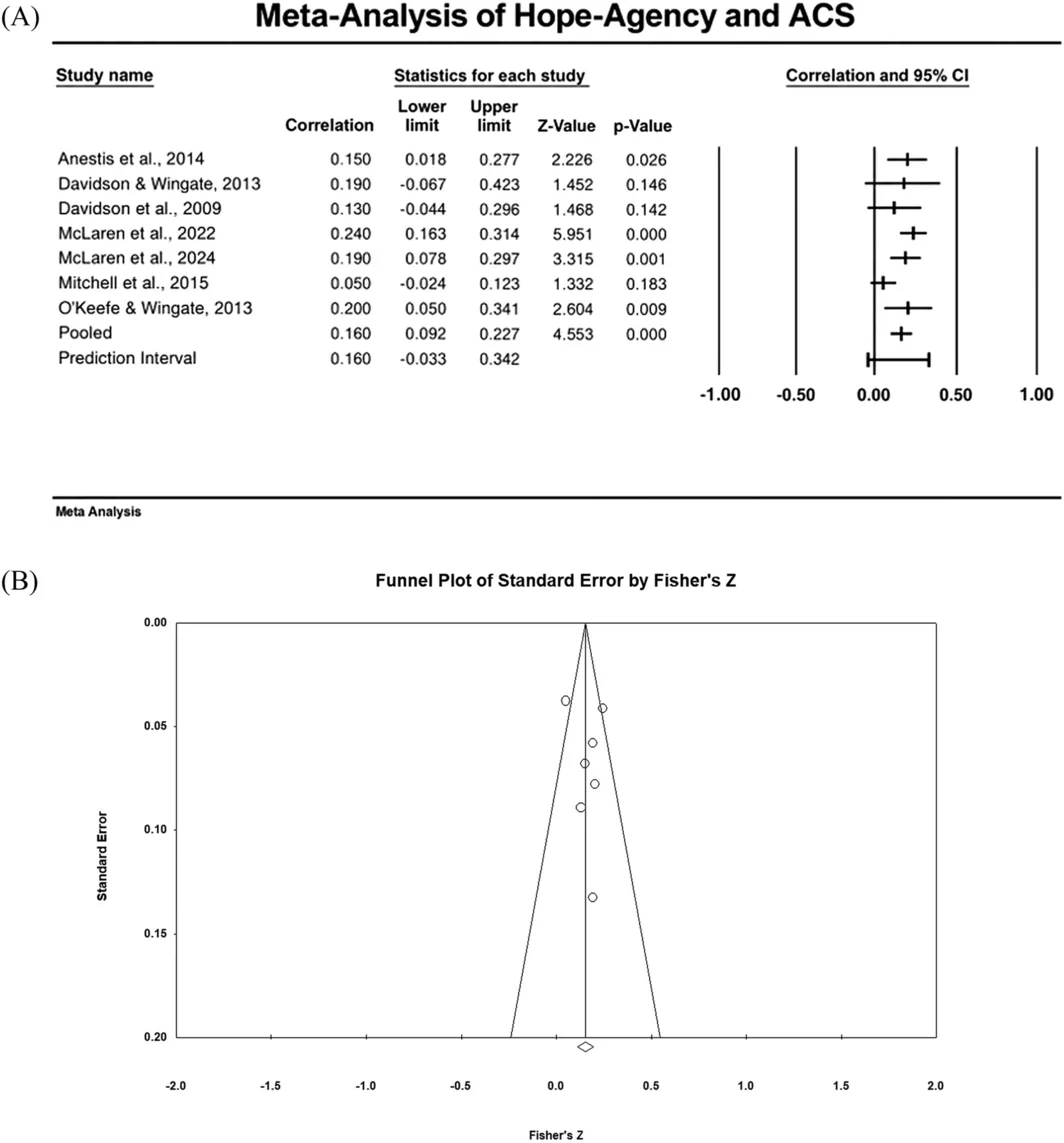
Results for Hope Agency. Top panel (A) is the forest plot of the association between hope agency and acquired capability for suicide (ACS). Each row represents an individual study association. Points indicate correlation coefficients, horizontal lines represent 95% confidence intervals, and the pooled estimate at the bottom reflects the overall meta‐analytic effect. The bottom panel (B) is the funnel plot assessing publication bias in the association between hope agency and acquired capability for suicide. Each point represents a study examining the association between total hope and acquired capability for suicide. Effect sizes are plotted as Fisher's *z* against standard error, with the vertical line indicating the pooled effect estimate.

There was significant heterogeneity across studies (*Q*(6) = 13.50, *p* = 0.04, *I* ^*2*^ = 55.56%). The 95% prediction interval [−0.033 to 0.342] suggests that the true effect in future studies may range from negligible to moderately positive, reflecting the possible presence of study moderators. Age significantly moderated the relationship between hope agency and acquired capability (z = 2.99, *Q*(1) = 8.95, *p* = 0.003), with stronger associations observed in samples with higher mean age. After accounting for age, residual heterogeneity was non‐significant (*Q*(5) = 4.55, *p* = 0.474, *I* ^2^ = 0%). Race (*z* = −0.33, *Q*(1) = 0.11, *p* = 0.74) and sex (*z* = 0.52, *Q*(1) = 0.27, *p* = 0.60) were not significant moderators.

## 4 Discussion

To our knowledge, this is the first meta‐analysis to quantify the association between hope and the acquired capability for suicide. Our findings build on the previous qualitative review (Ropaj 2023), and indicate that hope, including both agency and pathways, showed small, positive, and significant pooled associations with acquired capability for suicide. Although hope has long been theorized as an important protective factor for suicide, these findings suggest that hope is associated with higher acquired capability for suicide, which may unexpectedly increase rather than decrease suicide risk. These findings align with theory, as Snyder (1994, 2002) conceptualizes goal pursuit without assigning value to whether those goals are adaptive or maladaptive (Snyder 2002). As such, it may offer important insight into the complex processes underlying the progression from suicidal desire to suicidal action.

Snyder (1994, 2002) theorized that low pathways and higher agency may impart the greatest risk for suicide. Our findings conflict with this theory and suggest that pathways and agency relate similarly to acquired capability. However, the implications of pathways and agency may depend on whether suicide is conceptualized as a goal itself or as a pathway toward another goal, such as reducing suffering. If suicide becomes a salient goal, greater pathways thinking could facilitate the generation of multiple routes toward that goal, while greater agency could provide the motivation to pursue it. From this perspective, the consequences of hope may depend not only on an individual's capacity for goal pursuit, but also on the goals toward which agency and pathways are directed. The mechanisms for how and why hope pathways and hope agency relate positively to acquired capability are not yet understood. Hope pathways may relate to acquired capability by enhancing problem‐solving and planning related to overcoming obstacles, including those involved in suicide risk; whereas, hope agency may relate to acquired capability through motivational drive (Grewal and Porter 2007). For example, clinical observations suggest that individuals experience a surge in mood, energy, or motivation just prior to a lethal attempt, particularly during early recovery from depression (American Psychiatric Association 2003; Joiner et al. 2004; Joiner 2005; Snyder 1994, 2002). Hope pathways and agency may relate similarly to acquired capability, but the mechanisms of action may differ. It is our hope that this review will catalyze future research examining the mechanisms underlying this relationship.

The included studies were cross‐sectional, precluding determination of causal direction. Previous work showing hope as a predictor of acquired capability (Davidson et al. 2010; Mitchell et al. 2015; Wagshul 2019) suggests that this association reflects a directional process in which elevated hope contributes to the development or strengthening of acquired capability. However, it is also possible that acquired capability influences hope, such that acquired capability reduces fear barriers to goal pursuit, or that hope and acquired capability could be influenced by a third factor, such as death anxiety (Chopik 2016; Russac et al. 2007; Sinoff 2017) or distress tolerance (Anestis et al. 2014). In addition, early traumatic experiences (Joiner 2005; Smith and Cukrowicz 2010) may also impact the development of both acquired capability and hope (Baldini et al. 2025; Munoz et al. 2018; Snyder 1994, 2002), warranting further investigation into who may be at greater risk for suicide. Future research should prioritize rigorous prospective and experimental designs to determine causal direction and identify modifiable, clinically relevant mechanisms with potential to reduce suicide risk.

Variability across the associations differed. The associations with total hope and pathways showed little to no heterogeneity. However, the association between acquired capability and agency showed significant heterogeneity. This heterogeneity was partially explained by age, such that the association was stronger in samples with older mean age. This could mean that the motivational component of hope may be an important and surprising marker for suicide risk in older adults. Social, functional, and health‐related losses in later life may increase perceived helplessness and reduce quality of life, narrowing perceived pathways to valued goals and increasing the likelihood that suicide is considered a goal‐directed option (Sadek et al. 2024). Older adults may also experience a narrowing of personally salient goals as opportunities, resources, or energy for goal pursuit change with age. A more restricted goal structure could theoretically heighten risk if high hope becomes increasingly directed toward suicide as a salient goal. Importantly, age variability was largely limited across included studies, reducing confidence in this effect and necessitating replication, as this age effect may be driven by study‐specific differences. Older adults are at elevated suicide risk due to higher method lethality, greater likelihood of death on a first attempt, and recent increases in suicide rates (Van Orden et al. 2012; Sadek et al. 2024; Centers for Disease Control and Prevention, National Center for Health Statistics 2023), making them a priority population for suicide prevention.

Race and sex were not significant moderators of between‐study variability for agency or pathways. Most studies included predominantly White samples, constraining the ability to meaningfully examine race as a moderator; this should be studied more fully in the future. Findings for sex suggest that, although there are mean‐level differences across sex (i.e., males report higher acquired capability and engage in more lethal suicidal behavior; Nock, Borges, Bromet, Cha, et al. 2008; Witte et al. 2012), sex does not alter the strength of the association between agency and acquired capability. This suggests that the underlying relationship may operate similarly across males and females. From a policy and intervention standpoint, these results may support the broader applicability of hope‐informed prevention strategies, while still recognizing the importance of addressing sex‐based disparities in suicide risk at structural and contextual levels.

There are several important next steps to advance research in this area. First, the current literature relies primarily on cross‐sectional data. An important next step is to invest in longitudinal and experimental designs to establish temporal precedence and infer causal relationships. Second, although acquired capability is theorized to comprise two components, fearlessness about death and pain tolerance, the measures used across studies were insufficient to examine differences in the relationships across these components. The ACSS (Van Orden et al. 2008) was originally developed to assess acquired capability broadly but has been criticized for lacking a clear factor structure and psychometric rigor (Ribeiro, Witte, et al. 2014; Rogers et al. 2021). The ACSS‐FAD, a revised version with stronger psychometric properties, assesses only fearlessness about death, omitting pain tolerance entirely (Ribeiro, Witte, et al. 2014). Our results likely better reflect associations with fearlessness about death, rather than pain tolerance. A key next step is to measure both components of acquired capability to directly test for differences across these components. In addition, as conceptualizations of suicide capability continue to evolve, examining the relationship between hope and related constructs, such as “practical capability” (Klonsky and May 2015) and “perceived ability to die by suicide” (Robison et al. 2025), may provide a more comprehensive understanding of the complex relationship between hope and suicide capability. Finally, all included studies measured trait, rather than state, hope. Trait hope captures an individual's enduring tendency to engage in goal‐directed thinking, whereas state hope reflects a more immediate, situational mindset regarding one's ability to pursue and achieve goals (Snyder et al. 1996). State hope can fluctuate over time and may be especially relevant during periods of crisis when suicide risk is elevated. Emerging research has emphasized that an individual's capability for suicide can dynamically increase during acute psychological states, facilitating the ability to act on suicidal thoughts (Law and Anestis 2021; Ribeiro, Silva, et al. 2014; Smith and Cukrowicz 2010). Temporary reductions in state hope could erode resilience, while temporary surges in goal‐directed thinking might inadvertently support movement toward suicidal action. This is a key question to answer in future work in order to better link hope with acquired capability in critical moments.

The current findings should be considered within the context of their limitations.

First, included articles were mostly limited to Western samples and may not generalize to other cultures or countries. Second, inconsistent reporting and conflating of sex and gender may mask differences across groups and limit generalizability to others, including those with non‐binary or transgender identities. Finally, the study may have been underpowered due to few studies meeting inclusion criteria; thus, null results may be subject to Type II error. These findings should be interpreted cautiously given the limited number of included studies; however, the relatively consistent effect sizes across studies provide some degree of confidence in the observed pattern of results.

## 5 Conclusion

In conclusion, this meta‐analysis found a small but significant positive pooled association between hope and acquired capability for suicide, with similar effects across agency and pathways. Although hope is typically linked to adaptive psychological outcomes (Corrigan and Schutte 2023; Murphy 2023), these findings suggest a potential exception: hope may increase suicide risk by enhancing acquired capability for suicide. This possibility aligns with hope theory's value‐neutral stance on goal pursuit, which does not distinguish between adaptive and maladaptive goals (Snyder 2002), and with ideation‐to‐action frameworks that emphasize factors facilitating the transition from suicidal thoughts to behavior (Klonsky and May 2015). Although effect sizes were small, they may hold clinical relevance, particularly for individuals at elevated risk for suicide. The limited attention given to this relationship highlights a need for more nuanced clinical assessment and intervention, as increasing hope, without strengthening adaptive goals or reasons for living, may be ill‐advised in some contexts and may depend on the presence of adaptive goals or reasons for living. Future research should prioritize longitudinal and experimental designs to establish temporal precedence, utilize comprehensive measures of acquired capability, examine distinct hope components, and assess state hope.

## Author Contributions

**Timothy B. Salguero:** conceptualization, investigation, methodology, formal analysis, writing – original draft, writing – review and editing. **Lindsey Fisher‐Fox:** formal analysis, writing – review and editing. **Julia L. Smith:** writing – original draft, writing – review and editing. **Yancey Sizemore:** investigation, writing – review and editing. **Kevin L. Rand:** supervision, writing – review and editing. **Melissa A. Cyders:** supervision, methodology, writing – review and editing.

## Ethics Statement

Data from this meta‐analysis were obtained from peer‐reviewed publications and reports that were presumed to have undergone appropriate ethical review according to local regulations. No new data were collected, and no identifiable information was used. Therefore, additional ethics approval and individual informed consent were not required for this study.

## Conflicts of Interest

The authors declare no conflicts of interest.

## Data Availability

The data that support the findings of this study are available from the corresponding author upon reasonable request.
